# Polymorphic Amplified Typing Sequences and Pulsed-Field Gel Electrophoresis Yield Comparable Results in the Strain Typing of a Diverse Set of Bovine *Escherichia coli* O157:H7 Isolates

**DOI:** 10.1155/2012/140105

**Published:** 2012-08-07

**Authors:** Indira T. Kudva, Margaret A. Davis, Robert W. Griffin, Jeonifer Garren, Megan Murray, Manohar John, Carolyn J. Hovde, Stephen B. Calderwood

**Affiliations:** ^1^Food Safety and Enteric Pathogens Research Unit, National Animal Disease Center, Agricultural Research Service, U.S. Department of Agriculture, Ames, IA 50010, USA; ^2^Department of Microbiology, Molecular Biology and Biochemistry, University of Idaho, Moscow, ID 83843, USA; ^3^Department of Veterinary Microbiology and Pathology, College of Veterinary Medicine, Washington State University, Pullman, WA 99164, USA; ^4^Division of Infectious Diseases, Massachusetts General Hospital, Boston, MA 02114, USA; ^5^Department of Epidemiology, Harvard School of Public Health, Boston, MA 02115, USA; ^6^Department of Medicine, Harvard Medical School, Boston, MA 02115, USA; ^7^Pathovacs Inc., Ames, IA 50010, USA; ^8^School of Food Sciences, University of Idaho, Moscow, ID 83843, USA; ^9^Department of Microbiology and Immunobiology, Harvard Medical School, Boston, MA 02115, USA

## Abstract

Polymorphic amplified typing sequences (PATS), a PCR-based *Escherichia coli* O157:H7 (O157) strain typing system, targets insertions-deletions and single nucleotide polymorphisms at *Xba*I and *Avr*II restriction enzyme sites, respectively, and the virulence genes (*stx1*, *stx2*, *eae*, *hlyA*) in the O157 genome. In this study, the ability of PATS to discriminate O157 isolates associated with cattle was evaluated. An in-depth comparison of 25 bovine O157 isolates, from different geographic locations across Northwest United States, showed that about 85% of these isolates shared the same dendogram clade by PATS and pulsed-field gel electrophoresis (PFGE), irrespective of the restriction enzyme sites targeted. The Pearson's correlation coefficient, *r*, calculated at about 0.4, 0.3, and 0.4 for *Xba*I-based, *Avr*II-based and combined-enzymes PATS and PFGE similarities, respectively, indicating that these profiles shared a good but not high correlation, an expected inference given that the two techniques discriminate differently. Isolates that grouped differently were better matched to their locations using PATS. Overall, PATS discriminated the bovine O157 isolates without interpretive biases or sophisticated analytical software, and effectively complemented while not duplicating PFGE. With its quick turnaround time, PATS has excellent potential as a convenient tool for early epidemiological or food safety investigations, enabling rapid notification/implementation of quarantine measures.

## 1. Introduction

*Escherichia coli *O157:H7 (O157) causes an estimated 63,153 domestically acquired foodborne illnesses, 2,138 hospitalizations and 20 deaths annually in the United States [1–7]. Although a 44% decline in O157 cases was reported for the year 2010, over the past six years at least 13 different multistate O157 outbreaks have occurred, many of which have had a direct link to beef or produce possibly contaminated with manure [6, 7]. In fact, with cattle being the primary reservoir for this human pathogen [2–4], most human infections occur through food sources that are cattle derived (undercooked hamburger) or contaminated by cattle feces, such as salad vegetables, water, apple cider, and unpasteurized milk. With the current mechanization and globalization trends in food production and distribution, the need to monitor produce for foodborne pathogens such as O157 continues to remain critical to the prevention of extensive outbreaks, as is rapid epidemiological surveillance to identify and eliminate potential sources from the food chain. 

Pulsed-field gel electrophoresis (PFGE) is the bacterial strain typing method of choice, regularly used by diagnostic and epidemiological laboratories to type O157 strains. To overcome the drawbacks of standard PFGE methodology, several modifications have been implemented that seek to address issues of, restriction enzyme inhibition, DNA degradation, variations in electrophoretic patterns between gels, improper resolution of digested DNA, subjective interpretation of these patterns even with sophisticated pattern-recognition computer software, and most importantly to decrease the turnaround time from 3 to 4 days to within 24 h, so data can be made available in a timely manner [8–12]. Even with all the modifications, it has been noted that single-restriction enzyme PFGE gives a poor measure of genetic relatedness as it does not resolve the entire repertoire of DNA fragments generated following restriction digestion [13]. 

Consequently, this has led to the incorporation of other genome-sequence-based techniques, such as multilocus sequence typing (MLST) and/or multilocus variable-number tandem repeat analysis (MLVA), either in conjunction with PFGE or by themselves, to type O157 isolates. However, even these methodologies cannot speed up the process as they rely primarily on generation of sequencing quality DNA, analysis of multiple genes or distances between tandem repeat sequences, which require complex instrumentation, and interpreting software [14, 15]. Hence, all these techniques would be useful in detailed, comprehensive analysis for followup cross-referencing, and banking purposes, rather than being the “first response” tools to rapidly sort out linked and unlinked cases/sources in an outbreak situation. 

In previous studies, a touchdown PCR-based O157 strain typing system that incorporated polymorphisms at the *Xba*I- and *Avr*II-restriction sites, and amplified four virulence genes in the O157 genome was standardized against 46 O157 isolates from different sources and outbreaks [16–18]. This system termed the polymorphic amplified typing sequences (PATS) was not only able to provide a DNA fingerprint but also provide virulence profiles of the examined O157 isolates. PATS was less discriminatory when only one of the restriction enzyme sites was targeted but in the combination indicated above, PATS matched related isolates better than PFGE while differentiating between the unrelated isolates [16–18]. 

In this study, we decided to evaluate PATS against a diverse set of bovine O157 isolates and compare the profiles generated, with the PFGE patterns for the same, at length. For this, we targeted the same combinations of restriction enzyme sites. Although PATS directly sorts the polymorphisms at the restriction enzyme sites, and PFGE analyzes the DNA fragments generated as a result of these polymorphisms, we wanted to identify the degree of similarity between the two techniques and also ascertain if PATS would continue to maintain its ability to relate/discriminate bovine isolates as it did for human isolates in earlier studies [18]. 

## 2. Materials and Methods

### 2.1. Bacteria

Twenty-five O157 bovine isolates from various farms along the northwest region of United States (Idaho, Washington, and Oregon states) were obtained from collections maintained at the Field Disease Investigation Unit, College of Veterinary Medicine, Washington State University, Pullman, WA. The identification code used for each of these isolates is as indicated in Table 1.

### 2.2. PATS

PCR was done using conditions and primers as described previously [16–18]. Briefly, colony lysate of each O157 strain was tested against individual primer pairs, using the hot start PCR technique [19] in combination with a touchdown PCR profile [20]. To create this profile, an amplification segment of 20 cycles was set where the annealing temperature started at 73°C to touchdown at 53°C at the end of those cycles. Then, another amplification segment of 10 cycles was set, using the last annealing temperature of 53°C. Each reaction was done in triplicate to confirm profiles generated. Primer pairs targeting the 8 polymorphic *Xba*I- and 7 polymorphic *Avr*II-restriction enzyme sites, and the four virulence genes encoding the Shiga toxin 1 (*stx*_1_), shiga toxin 2 (*stx*_2_), Intimin-*γ* (*eae*), and hemolysin-A (*hlyA*), were used [16–18]. PCR reactions were purified using the QIAquick PCR purification kit (Qiagen, Valencia, Ca.), and all reactions, except for those amplifying virulence genes, were digested with the appropriate restriction enzyme (New England Biolabs, Beverly, Ma.) to confirm the presence of the restriction site within amplicons prior to resolution on a 4% agarose gel.

Presence or absence of amplicons was recorded as before [17, 18]. Briefly, for the virulence genes, the presence of an amplicon was recorded as “1” (as these lacked either of the restriction enzyme sites being tested) and “0” for the absence of an amplicon. For PCR targeting the polymorphic *Xba*I restriction sites, presence of an amplicon was recorded as a “2” (as all amplicons could be digested into 2 fragments following enzymatic cleavage by the *Xba*I restriction enzyme) and as “0” in the absence of an amplicon. Likewise, for PCR targeting the *Avr*II restriction site, presence of an amplicon was recorded as “1” if the amplicon had no *Avr*II site, “2” if the amplicon had an *Avr*II site that resulted in it being digested into 2 fragments following enzymatic cleavage by the *Avr*II restriction enzyme and “0” in the absence of an amplicon [18]. 

### 2.3. PFGE

Standard PFGE methods were used to analyze the 25 bovine isolates as previously described [13, 18]. Briefly, the genomic DNA of each isolate was embedded in separate agarose plugs and digested at 37°C for 2 h with 30 U of *Xba*I or *Bln*I (*Avr*II) (Gibco BRL, Grand Island, N.Y.) per plug. The plugs were loaded onto a 1% agarose-tris buffer gel (SeaKem Gold Agarose; BioWhittaker Molecular Applications, Rockland, Maine), and PFGE was performed with a CHEF Mapper XA apparatus (Bio-Rad Laboratories, Hercules, Calif.). DNA was electrophoresed for 18 h at a constant voltage of 200 V (6 V/cm), with a pulse time of 2.2 to 54.2 s, an electric field angle of 120°, and a temperature of 14°C, before being stained with ethidium bromide. Gel images were analyzed using Bionumerics (Applied Maths, Saint-Martens-Latem, Belgium). 

### 2.4. Data Analysis

(i) PATS dendograms: dendograms were constructed by coding molecular data as described previously [16–18]. Briefly, the presence or absence of each of the 15 amplicons (representing 8 polymorphic *Xba*I and 7 polymorphic *Avr*II sites) was coded as a dichotomous variable. Characters representing gain or loss of a restriction site recognized by *Avr*II were weighted to reflect the increased probability of losing a site than gaining one; however, such weighting had no impact on the resulting dendogram. Trees were constructed using the unweighted pair-group method with arithmetic means (UPGMA) option in the phylogenetic analysis using parsimony (PAUP; Sinauer Associates, Inc., Publishers, Sunderland, Ma.). (ii) PFGE dendograms: Dendograms were constructed using UPGMA cluster analysis based on Dice coefficients performed in Bionumerics, for *Xba*I- and *Avr*II-based PFGE patterns [21]. Combined Dice coefficients were then used to generate the *Xba*I and *Avr*II-combined-PFGE dendogram. (iii) Similarities: Bionumerics was used to determine Dice similarity coefficients for PFGE banding patterns as described previously, using the formula, 2 *n*/*a* + *b*, where *n* = number of matching bands and a + b = total number of bands (matching and nonmatching) being compared between a pair of O157 isolates [13, 21]. For PATS, this coefficient was manually derived for each isolate pair using the modified formula, {2 × the number of concordant markers} ÷ {total number of markers being compared}. The total number of markers being compared between a pair of O157 isolates was 12 for *Xba*I-based PATS, and 11 for *Avr*II-based PATS, including the virulence genes. The Dice similarity coefficients for PFGE and PATS were then used to calculate Pearson's correlation coefficients as shown in the scatter plots.

## 3. Results

### 3.1. PATS Screening of the 25 Bovine O157 Isolates

**Figure 1:**
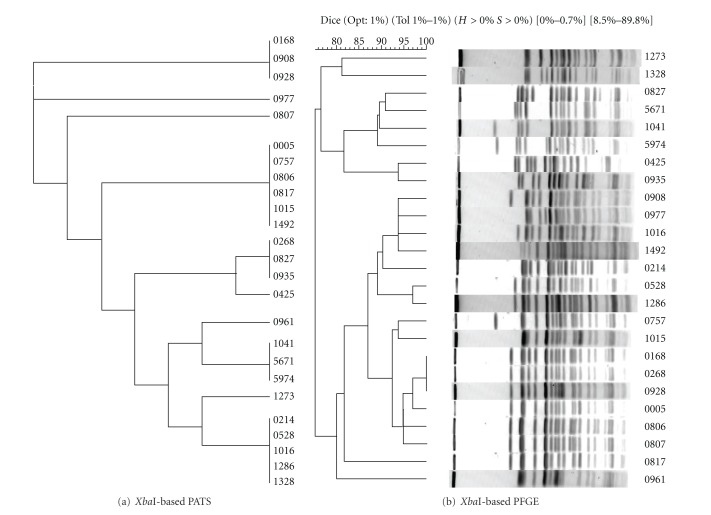
Analysis of relatedness between 25 bovine O157 isolates. (a) Dendogram for *Xba*I-based PATS profiles was constructed using the UPGMA option in the phylogenetic analysis using parsimony (PAUP; Sinauer Associates, Inc., Publishers, Sunderland, Ma.). (b) Dendogram for *Xba*I-based PFGE profiles was constructed using the UPGMA cluster analysis based on Dice coefficients performed in Bionumerics (Applied Maths, Saint-Martens-Laatem, Belgium). Percent tolerance used is shown above the dendogram.

**Figure 2:**
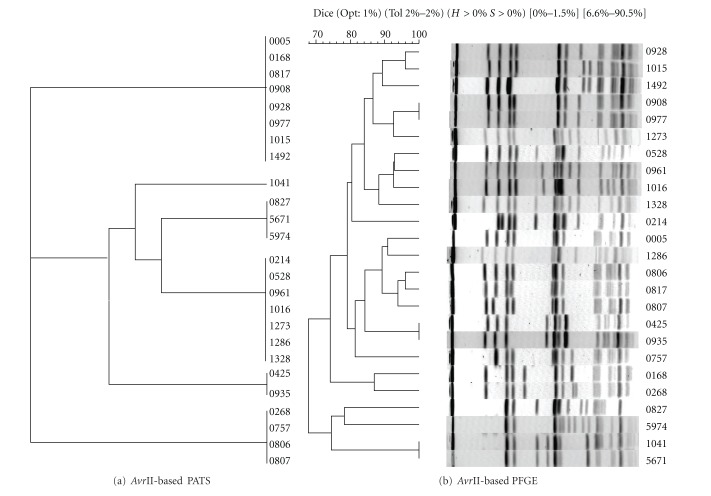
Analysis of relatedness between the 25 bovine O157 isolates. (a) Dendogram for *Avr*II-based PATS profiles was constructed using the UPGMA option in the phylogenetic analysis using parsimony (PAUP; Sinauer Associates, Inc., Publishers, Sunderland, Ma.). (b) Dendogram for *Avr*II-based PFGE profiles was constructed using the UPGMA cluster analysis based on Dice coefficients performed in Bionumerics (Applied Maths, Saint-Martens-Laatem, Belgium). Percent tolerance used is shown above the dendogram.

**Figure 3:**
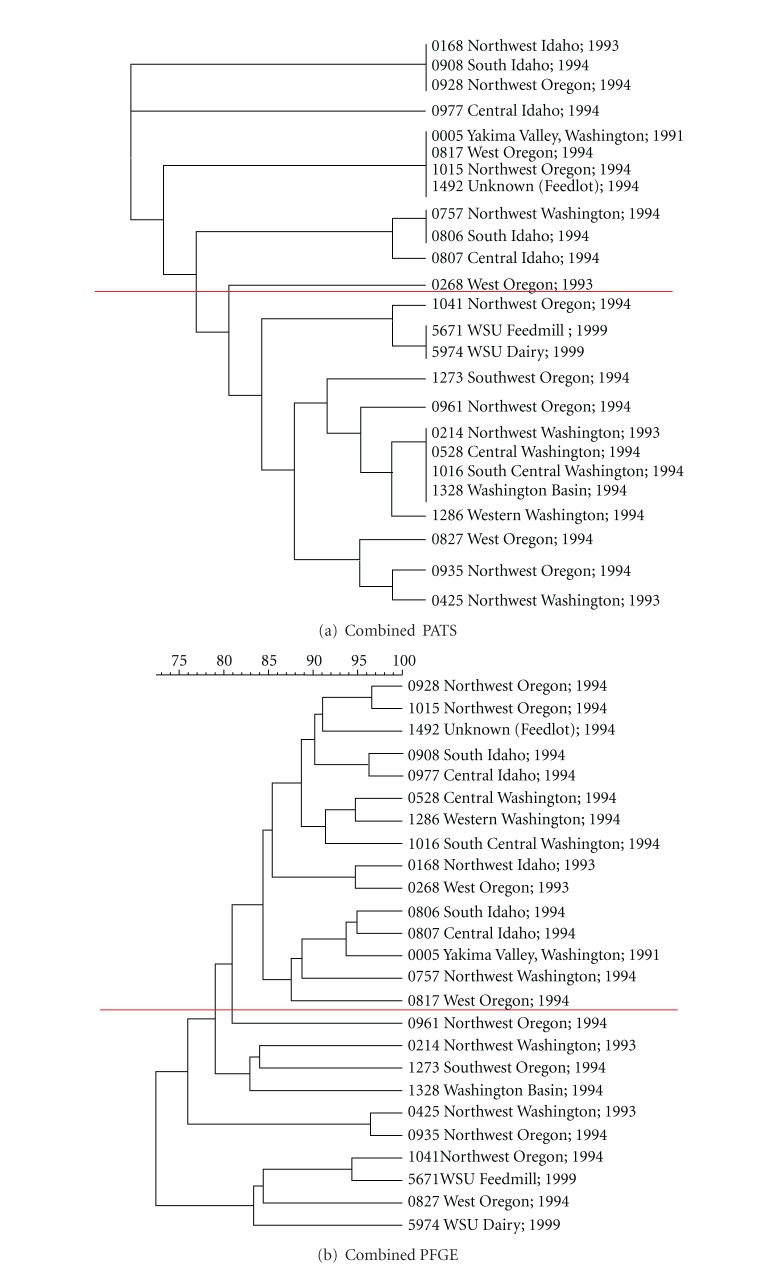
Analysis of relatedness between the 25 bovine O157 isolates. (a) Dendogram for combined-PATS (*Xba*I-, *Avr*II- and virulence genes-based) profiles was constructed using the UPGMA option in the phylogenetic analysis using parsimony (PAUP; Sinauer Associates, Inc., Publishers, Sunderland, Ma.). (b) Dendogram for combined-PFGE resulting from cluster analysis of combined Dice coefficients from PFGE following digestion with *Xba*I and PFGE following digestion with *Avr*II. Red line on each dendogram delineates isolates similarly grouped by combined-PATS and combined-PFGE.

 All O157 isolates were analyzed for polymorphic *Xba*I- and *Avr*II-restriction sites, along with virulence genes. Independent of each other, *Xba*I-based PATS generated 10 different profiles, while *Avr*II-based PATS generated 6 different profiles (Tables 2(a) and 2(b)). However, in combination along with the four virulence genes, PATS analysis resulted in 15 distinct profiles demonstrating an increase in discrimination as observed previously (Table 3) [18]. These distinct profiles were clustered into smaller, related clades in the dendograms generated in PAUP. As shown in Figures 1(a), 2(a), and 3(a), the *Xba*I-based, *Avr*II-based, and combined PATS generated 5, 3, and 7 clades, respectively. Interestingly, five PATS profiles, 1, 2, 3, 4, and 11 (Table 3), were identical to the PATS profiles 19, 2, 18, 16, and 8, respectively, observed in a previous analysis of 46 unrelated O157 isolates associated with human disease [18], which may be reflective of the clonality of O157 isolates despite its divergence into multiple strain types [22]. 

### 3.2. PFGE Analysis of the 25 Bovine O157 Isolates

As shown in Figures 1(b), 2(b), and 3(b), PFGE analysis of the 25 bovine O157 isolates yielded complex genomic DNA electrophoresis patterns. Whether the isolates were grouped traditionally based on band differences (identical, closely related with 1–3 band differences, more distantly related with 4–6 band differences) (data not shown), or grouped based on Dice similarity coefficients, the dendograms generated in Bionumerics indicated a high similarity among the isolates. Using the latter configurations, *Xba*I-based PFGE generated 6 clades from 23 different DNA banding patterns, *Avr*II-based PFGE generated 7 clades from 22 different DNA banding patterns, and combined PFGE generated 9 clades. 

### 3.3. PATS Has Good Correlation with PFGE While Maintaining Its Distinctive Discriminating Features

**Figure 4:**
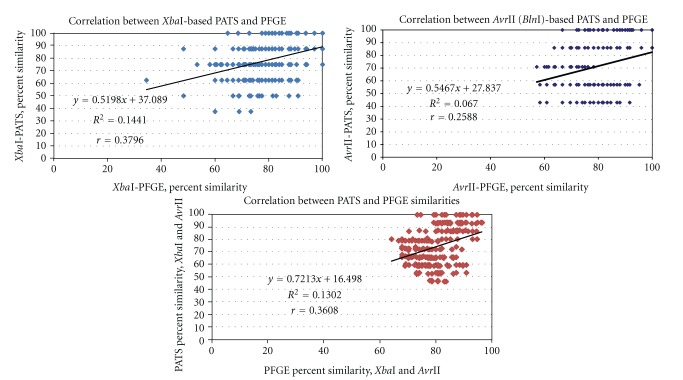
Scatter plots comparing PATS and PFGE profiles generated for 25 bovine O157 isolates. The derivation of the Pearson correlation coefficient is shown within each graph.

Comparison of the dendograms generated showed that about 85% of the bovine O157 isolates formed similar groups by PATS and PFGE, irrespective of whether it was *Xba*I-based (84% similar groups), *Avr*II-based (84% similar groups), or based on a combination of enzymes (88% similar groups). However, the inherent differences between the two techniques was reflected when the Dice similarity coefficients were subjected to Pearson's correlation coefficient analysis. The Pearson's correlation coefficient, *r*, was calculated at about 0.4, 0.3, and 0.4 for *Xba*I-based, *Avr*II-based and combined PATS and PFGE similarities, respectively (Figure 4). This clearly indicated that these profiles shared a good if not high correlation. This may be reflective of the two techniques assessing the same restriction site polymorphisms in a different manner; PATS directly ascertains the presence/absence/other variations at these sites, while PFGE evaluates the resulting variations in the number and sizes of the genomic DNA fragments generated post-digestion with the same restriction enzymes. 

A more positive correlation was observed for the *Xba*I-based, and *Xba*I and *Avr*II-combined-PFGE and PATS profiles than for the *Avr*II-based profiles, suggesting that perhaps the latter was more discriminatory. In fact, polymorphisms at the *Avr*II restriction sites and the presence/absence of virulence genes increased the discriminatory ability of PATS. Although we cannot rule out the inability to resolve ambiguous patterns due to comigrating bands or incompletely digested spurious bands by PFGE, this observation with PATS lends support to some of the discrimination seen with PFGE as well [17, 18]. Thus, the two typing techniques seem to complement each other while maintaining their own discriminative features.

## 4. Discussion

The goal of this study was to compare the results of *Xba*I-based-, *Avr*II-based-, and combined-PATS with similar analyses done using PFGE, on 25 bovine O157 collected from different geographic locations to determine if these two strain typing techniques would relate and discriminate between bovine isolates in a similar manner as previously reported with human isolates. *Xba*I-based, *Avr*II-based, and combined PATS generated 5, 3, and 7 clades, respectively, reflecting the clonality of O157 (Figures 1(a) and 2(a)). A similar tendency was observed with *Xba*I-based (6 clades), *Avr*II-based (7 clades), and combined (9 clades) PFGE profiles based on the Dice coefficient similarities (Figures 1(b) and 2(b)). Clades generated with combined-PATS grouped the majority of isolates from Washington State and Idaho State in separate clusters interspersed with isolates from Oregon State (Figure 3(a)). While combined-PFGE-generated clades did not distribute the isolates in the exact manner as combined-PATS, about 85% of the isolates maintained a similar distribution (Figure 3(b)). As this was a random study of isolates, we were unable to determine if there was any transfer of animals, feed or other farm related goods between Oregon and the other 2 states that may have caused the O157 isolates to be closely related [21]. However, in an epidemiological situation this would be a reasonable cause to verify such an exchange. 

PFGE is currently the standard strain typing technique used by various epidemiological and diagnostic laboratories to estimate the relatedness of outbreak or nosocomial O157 and other bacterial isolates [12, 23]. Compared to other strain typing methodologies being used, PFGE does provide relatively distinctive profiles for strains in several serotypes making it a popular strain typing tool. However, challenges in using PFGE are well known especially, the improper digestion and resolution of DNA bands, comigration of similar sized DNA fragments and nonhomologous DNA, or changes in electrophoretic conditions or analytical software, resulting in “untypeable” or incorrect profiles that cannot be interpreted or compared [8–11, 13, 24]. These drawbacks have led to several measures to make the PFGE protocol more uniform across laboratories, along with suggestions to use additional restriction enzymes, speed up turnaround time [13, 23, 25–29], and use other DNA sequence-based typing systems in parallel to confirm the validity of observations made with PFGE. Yet these variations continue to impede streamlining this process. Assessing genetic relatedness in a timely manner is crucial to any epidemiological survey. PFGE and DNA-sequence-based typing systems rely on expensive instrumentation and software to interpret data, which make them more useful in detailed analysis and banking of pathogens. In the field, however, a rapid and straightforward technique with sufficient power to discriminate between isolates without technical and subjective biases would help track down sources and speed up the process of sorting out linked and unlinked cases/sources in an outbreak situation. Such a technique would need to complement PFGE and not duplicate it or render the process more cumbersome. 

In this study, both combined-PATS and PFGE had comparable discriminatory abilities, and the use of two restriction enzymes may have added to the observed similarities. O157 isolates that shared the same PATS group also fell into the same clade by PFGE. Some O157 isolates that fell into different groups/clades by the two techniques were better linked to their location using PATS which supports possible over-discrimination by PFGE. As seen previously [18], PATS was reliable, simple, user-friendly, and easy to perform and interpret in this instance as well. Because PATS is a PCR-based technique that directly addresses polymorphisms at restriction enzyme sites (Indels or SNPs), it eliminates the need for extensive electrophoresis, sequence analysis, and software to interpret results, thereby making it cost effective as well. PATS continued to maintain its high typeability and reproducibility as observed previously [18]. Based on all these observations, it appears that PATS would make an ideal “first response” epidemiological tool. We are in the process of evaluating the reliability of PATS in a “blind study” where the details of the O157 being typed will be withheld until the end of the study to ensure a typical field situation. We are also expanding application of PATS to other human pathogens of bovine origin such as, Shiga-toxin producing *Escherichia coli *(STECs), while seeking options to automate the process to further reduce the turnaround time to less than 6–8 hrs.

## Figures and Tables

**Table 1:** Summary of bovine *E. coli *O157:H7 isolates used in this study.

Isolates	Source	Location	Collection dates
5	Dairy cattle	Yakima Valley, Washington	7/22/1991
168	Dairy cattle	Northwest Idaho	6/7/1993
214	Dairy cattle	Northwest Washington	6/30/1993
268	Dairy cattle	West Oregon	8/3/1993
425	Dairy cattle	Northwest Washington	10/26/1993
528	Dairy calf	Central Washington	4/21/1994
757	Dairy cattle	Northwest Washington	6/21/1994
806	Dairy cattle	South Idaho	7/5/1994
817	Dairy cattle	West Oregon	7/6/1994
807	Dairy cattle	Central Idaho	7/6/1994
827	Dairy cattle	West Oregon	7/11/1994
908	Dairy cattle	South Idaho	7/12/1994
928	Dairy cattle	Northwest Oregon	7/13/1994
935	Dairy cattle	Northwest Oregon	7/13/1994
961	Dairy cattle	Northwest Oregon	7/18/1994
977	Dairy cattle	Central Idaho	7/25/1994
1015	Dairy cattle	Northwest Oregon	7/25/1994
1016	Dairy cattle	South Central Washington	7/27/1994
1041	Dairy cattle	Northwest Oregon	8/1/1994
1273	Dairy cattle	Southwest Oregon	8/30/1994
1286	Dairy cattle	Western Washington	9/2/1994
1328	Dairy cattle	Washington Basin	9/13/1994
1492	Feedlot	Unknown	10/3/1994
5671	Mill feed	WSU^a^ Feedmill	1/5/1999
5974	Dairy cattle	WSU Dairy	9/1/1999

^a^WSU: Washington State University, Pullman, WA.

**Table:** (a)

PATS type^a^	PCR amplification and restriction digestion patterns of amplicons obtained using 8 PATS primer pairs^b^								Isolates^c^
	IK8	IK19	IK25	IK114	IK118	IK123	IKB3	IKB5
1	2	2	0	2	2	2	2	2	168, 908, 928,
2	0	2	0	2	2	2	2	2	977
3	2	2	0	2	2	2	0	2	5, 757, 806, 817, 1015, 1492
4	0	2	0	2	2	2	0	2	807
5	2	2	0	2	2	2	0	0	268, 827, 935
6	2	2	0	2	2	0	0	0	1041, 5671, 5974
7	2	2	0	0	2	2	2	0	1273
8	2	2	0	0	2	2	0	0	214, 528, 1016, 1286, 1328
9	2	2	0	2	0	2	0	0	425
10	0	2	0	0	2	0	0	0	961

**Table:** (b)

PATS type^a^	PCR amplification and restriction digestion patterns of amplicons obtained using 7 PATS primer pairs^d^							Isolates^c^
	IKNR3	IKNR7	IKNR10	IKNR12	IKNR16	IKNR27	IKNR33
1	2	2	2	2	2	2	2	5, 168, 817, 908, 928, 977, 1015, 1492
2	2	2	2	0	2	2	2	268, 757, 806, 807
3	2	2	2	2	1	2	2	1041
4	0	2	2	2	1	2	2	827, 5671, 5974
5	1	2	2	2	1	2	0	214, 528, 961, 1016, 1273, 1286, 1328
6	2	1	1	2	1	2	2	425, 935

^a^PATS types are designated arbitrarily with different numbers.

^b^Prefixes of each PATS primer pair A/B are indicated. 0: no amplicon; 2: amplicon with one *Xba*I site.

^c^Bovine *E. coli *O157:H7 isolates from different locations that fell within a given PATS type.

^d^Prefixes of each PATS primer pair A/B are indicated. 0: no amplicon; 1: amplicon without *Avr*II site; 2: amplicon with one *Avr*II site.

**Table 3:** Combined-PATS profiles for the 25 bovine O157 isolates.

	PCR amplification and restriction digestion patterns of amplicons obtained using 15 PATS—4 virulence gene primer pairs^b^																					
PATS type^a^	Polymorphic *Xba*I sites									Polymorphic *Avr*II sites							Virulence genes					Isolates^c^
	IK8	IK19	IK25	IK114	IK118	IK123	IKB3	IKB5	—	IKNR3	IKNR7	IKNR10	IKNR12	IKNR16	IKNR27	IKNR33	—	*stx*_1_	*stx*_2_	*eae*	*hlyA*	
1 (previous 19)^d^	2	2	0	2	2	2	2	2		2	2	2	2	2	2	2		1	1	1	1	168, 908, 928
2 (previous 2)	0	2	0	2	2	2	2	2		2	2	2	2	2	2	2		1	1	1	1	977
3 (previous 18)	2	2	0	2	2	2	0	2		2	2	2	2	2	2	2		1	1	1	1	5, 817, 1015, 1492
4 (previous 16)	2	2	0	2	2	2	0	2		2	2	2	0	2	2	2		1	1	1	1	757, 806
5	0	2	0	2	2	2	0	2		2	2	2	0	2	2	2		1	1	1	1	807
6	2	2	0	2	2	2	0	0		2	2	2	0	2	2	2		1	1	1	1	268
7	2	2	0	2	2	0	0	0		2	2	2	2	1	2	2		1	1	1	1	1041
8	2	2	0	2	2	0	0	0		0	2	2	2	1	2	2		1	1	1	1	5671, 5974
9	2	2	0	0	2	2	2	0		1	2	2	2	1	2	0		1	0	1	1	1273
10	2	2	0	2	2	2	0	0		0	2	2	2	1	2	2		0	1	1	1	827
11 (previous 8)	2	2	0	2	2	2	0	0		2	1	1	2	1	2	2		0	1	1	1	935
12	2	2	0	0	2	2	0	0		1	2	2	2	1	2	0		0	1	1	1	214, 528, 1016, 1328
13	2	2	0	2	0	2	0	0		2	1	1	2	1	2	2		0	1	1	1	425
14	2	2	0	0	2	2	0	0		1	2	2	2	1	2	0		0	1	1	0	1286
15	0	2	0	0	2	0	0	0		1	2	2	2	1	2	0		0	1	1	1	961

^a^PATS types are designated arbitrarily with different numbers.

^b^Prefixes of each PATS primer pair A/B and virulence gene primer pair F/R are indicated. 0**:** no amplicon; 1**:** amplicon without *Avr*II site; 2**:** amplicon with one *Xba*I or *Avr*II site.

^c^Bovine *E. coli *O157:H7 isolates from different locations that fell within a given PATS type.

^d^Identical profiles observed in previous study [18].
